# Influence of Plasma Atherogenic Index on Coronary Artery Disease Severity: Insights From a Large-Scale Cohort Study in China

**DOI:** 10.31083/RCM45510

**Published:** 2026-04-14

**Authors:** Xiang Wang, Wei Xu, Qirui Song, Zinan Zhao, Chenxi Xia, Yi Li, Yibo Xie, Jun Wang, Chenguang Yang, Zhenyan Zhao, Xuyang Meng, Fang Wang

**Affiliations:** ^1^Department of Cardiology, Beijing Hospital, National Center of Gerontology, Institute of Geriatric Medicine, Chinese Academy of Medical Sciences, 100730 Beijing, China; ^2^Emergency Center, Fuwai Hospital, State Key Laboratory of Cardiovascular Disease of China, National Center for Cardiovascular Diseases, National Clinical Research Center of Cardiovascular Diseases, Chinese Academy of Medical Sciences and Peking Union Medical College, 100037 Beijing, China; ^3^Hypertension Center, Fuwai Hospital, State Key Laboratory of Cardiovascular Disease of China, National Center for Cardiovascular Diseases of China, Chinese Academy of Medical Sciences and Peking Union Medical College, 100037 Beijing, China; ^4^Department of Pharmacy, Beijing Hospital, National Center of Gerontology, Institute of Geriatric Medicine, Chinese Academy of Medical Sciences, Beijing Key Laboratory of Assessment of Clinical Drugs Risk and Individual Application (Beijing Hospital), 100730 Beijing, China; ^5^Graduate School, Peking University Fifth School of Clinical Medicine, 100730 Beijing, China; ^6^Graduate School, Peking Union Medical College, Chinese Academy of Medical Science, 100730 Beijing, China; ^7^Department of Information Center, Beijing Hospital, National Center of Gerontology, Institute of Geriatric Medicine, Chinese Academy of Medical Sciences, 100730 Beijing, China; ^8^Department of Cardiology, Fuwai Hospital, National Center for Cardiovascular Diseases, Chinese Academy of Medical Sciences and Peking Union Medical College, 100037 Beijing, China; ^9^Clinical Trial Center, Beijing Hospital, National Center of Gerontology, Institute of Geriatric Medicine, Chinese Academy of Medical Sciences, 100730 Beijing, China

**Keywords:** coronary artery disease, diabetes mellitus, dyslipidemia, lipoproteins, triglycerides, high-density lipoproteins

## Abstract

**Background:**

The atherogenic index of plasma (AIP), calculated from triglyceride and high-density lipoprotein cholesterol levels, is associated with atherosclerosis and coronary artery disease (CAD). However, evidence concerning the impact of the AIP on CAD severity remains limited. This study aims to assess the correlation between AIP and the severity of CAD.

**Methods:**

This study included 19,929 hospitalized participants diagnosed with CAD. After excluding participants with missing data, aged >75 years, or diagnosed with chronic kidney disease or cancer, a total of 2561 individuals were included. The 2561 participants were divided into three AIP tertile groups: AIP1 (AIP <0.016, n = 854), AIP2 (0.016 ≤ AIP < 0.216, n = 853), and AIP3 (AIP ≥0.216, n = 854). In this study, CAD severity was determined by the count of coronary arteries exhibiting stenosis of 50% or greater. Multivessel CAD was defined as ≥50% stenosis in two or more major coronary arteries. The relationship between AIP and CAD severity was assessed using logistic regression models.

**Results:**

Results indicate that the AIP independently predicts CAD severity, with an odds ratio of 1.700 (95% confidence interval (CI): 1.160–2.491; *p* = 0.007). The AIP3 group demonstrated a significantly higher risk of multivessel CAD compared to the AIP1 group (odds ratio (OR), 1.441; 95% CI: 1.124–1.848; *p* = 0.004), particularly in patients without diabetes mellitus (OR, 1.421; 95% CI: 1.030–1.962; *p* = 0.033).

**Conclusions:**

The AIP was significantly associated with CAD severity, suggesting that it could be a convenient and valuable marker for severity stratification in patients with CAD in clinical practice.

## 1. Introduction

Coronary artery disease (CAD) is a significant global public health issue, contributing substantially to morbidity and mortality [1]. Systemic inflammation-induced atherosclerosis is the fundamental pathological mechanism of CAD [2]. Traditional lipid markers such as total cholesterol (TC), triglyceride (TG), and low-density lipoprotein cholesterol (LDL-C) are recognized predictors of CAD [3, 4]. Composite parameters such as TC/high-density lipoprotein cholesterol (HDL-C), TG/HDL-C, LDL-C/HDL-C, and non-HDL (TC minus HDL-C) have shown greater predictive value than individual lipid markers [5, 6]. The atherogenic index of plasma (AIP), introduced by Dobiásová and Frohlich in 2001, is calculated as log10 (TG/HDL-C) and effectively represents the balance between proatherogenic and antiatherogenic factors [7, 8]. Numerous studies have validated the AIP as an effective predictor of atherosclerosis and CAD [9, 10, 11, 12, 13].

A higher AIP has been shown to correlate with smaller LDL particle size, reflecting an increased proportion of small dense LDL particles [14]. According to published reports, sdLDL is more potent than LDL in eliciting atherogenic effects because of its susceptibility to oxidation and its propensity to stimulate the production of foam cells [14, 15, 16, 17]. However, the measurement technology for assessing sdLDL is complex and costly [18]. AIP has emerged as a convenient and economical surrogate marker for sdLDL [19, 20].

Coronary artery disease severity, particularly multivessel CAD, is strongly associated with adverse clinical outcomes. Although recent studies have increasingly associated AIP with CAD prognosis [9, 20, 21], research on its impact on CAD severity remains scarce. Evidence on the correlation between AIP and coronary artery stenosis severity across varying diabetes statuses is lacking. This research aimed to determine how AIP relates to CAD severity among a large cohort of patients and whether this relationship is influenced by the presence of diabetes.

## 2. Materials and Methods

### 2.1 Study Design and Population

**Fig. 1.**
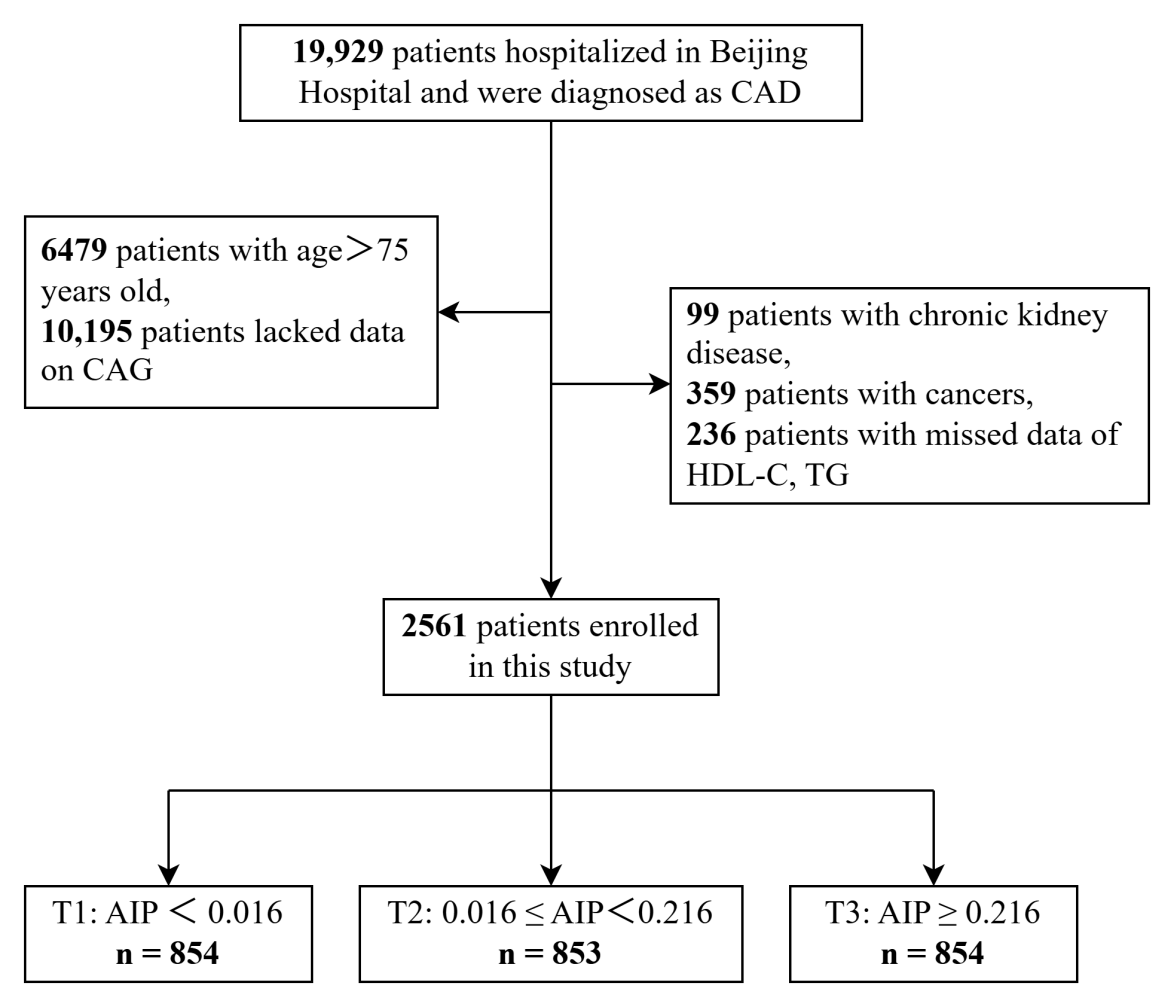
**Flowchart of the study population**. CAD, coronary artery disease; CAG, coronary angiography; HDL-C, high-density lipoprotein cholesterol; TG, triglyceride; AIP, atherogenic index of plasma.

The study adhered to the Declaration of Helsinki and received approval from the Beijing Hospital ethics committee. All the participants provided written informed consent. This cohort study included 19,929 participants diagnosed with CAD and hospitalized at Beijing Hospital between January 2016 and December 2021. All the patients were diagnosed with CAD by experienced clinicians according to clinical symptoms combined with CT angiography (CTA) or coronary angiography (CAG). Those aged >75 years (n = 6479), lacking CAG data (n = 10,195), with chronic kidney disease (n = 99), with cancer (n = 359), and without HDL-C and TG data (n = 236) were excluded, as shown in Fig. 1.

Finally, 2561 participants were included in the present statistical analysis. We divided the 2561 participants into three groups according to the AIP tertiles: the AIP1 (AIP <0.016; n = 854), AIP2 (0.016 ≤ AIP < 0.216; n = 853), and AIP3 (AIP ≥0.216; n = 854) groups.

### 2.2 Measurements and Definitions

The AIP was calculated using the formula log10 (TG/HDL-C). Fasting blood samples (≥8 hours) were collected from all participants. The detailed measurements of laboratory parameters are provided in the **Supplementary Material**. Body mass index (BMI) was determined by dividing weight in kilograms by height in meters squared. Clinical data were obtained from hospital records. Upon admission, systolic blood pressure (SBP), diastolic blood pressure (DBP), and heart rate were documented. Diabetes mellitus (DM) was characterized by fasting blood glucose (FBG) ≥7.0 mmol/L, 2-hour plasma glucose ≥11.1 mmol/L from an oral glucose tolerance test, glycated hemoglobin A1c (HbA1c) ≥6.5%, or a prior DM diagnosis. Participants without these criteria were considered non-DM.

The primary outcome was CAD severity. Experienced clinicians performed CAG through percutaneous femoral access, remaining blinded to the study protocol. Coronary artery disease severity was assessed by counting the coronary arteries exhibiting stenosis of 50% or greater [22]. CAD was considered single-vessel if only one major coronary artery had ≥50% stenosis, and multivessel if two or more arteries met this criterion.

### 2.3 Statistical Analysis

Statistical analyses in this study were conducted using SPSS Statistics (Version 27.0.1; IBM, Armonk, NY, USA), R (Version 4.0.3; R Foundation for Statistical Computing, Vienna, Austria), and SAS (Version 9.4; SAS Institute Inc., Cary, NC, USA). All *p* values were two-sided, with *p* < 0.05 indicating statistical significance.

Continuous variables are presented as mean ± standard deviation or median with interquartile range, while categorical variables are shown as counts (n) or percentages (%). For missing data, continuous variables, including SBP, DBP, heart rate (HR), estimated glomerular filtration rate (eGFR), and BMI, were imputed using the mean values. Baseline variables among AIP groups were compared using one-way ANOVA or the Kruskal–Wallis test as applicable. For variables comparing single-vessel and multivessel CAD, normality and variance homogeneity were evaluated. The independent sample *t*-test was applied to normally distributed variables with equal variance, while the Mann–Whitney U test was used for variables not meeting normality assumptions. The chi-square test was performed to compare categorical variables across the groups. Odds ratios (ORs) and 95% confidence intervals (CIs) for CAD severity in each AIP group were calculated using univariate and multivariate logistic regression models.

Three models were analyzed: Model 1 was unadjusted; Model 2 accounted for age and sex; Model 3 included additional adjustments for lifestyle factors, blood pressure, kidney function, BMI, and relevant medications, excluding those already considered in Model 2. Variables included in the multivariable regression were selected primarily on the basis of clinical experience and previously published literature [23].

## 3. Results

### 3.1 Baseline Characteristics

Key clinical characteristics across AIP tertiles and CAD severity subgroups are summarized below. The cohort included 2561 patients (mean age 63 ± 8 years), of whom 73.4% were male. Table 1 displays the baseline characteristics categorized by AIP tertiles. Compared with the AIP1 and AIP2 groups, the AIP3 group was more likely to include male and younger patients (all *p* < 0.05). Patients in the AIP3 group also had higher levels of FBG, BMI, LDL-C, TC, TG, and HbA1c but lower HDL-C levels (all *p* < 0.05). Moreover, the AIP3 group had higher proportions of tobacco and alcohol use, hypertension, antihypertensive drug use, and DM (all *p* < 0.05).

**Table 1.** **Baseline clinical and demographic characteristics stratified by AIP tertiles**.

Variables		Total	AIP1	AIP2	AIP3	*p* value
		(n = 2561)	(n = 854)	(n = 853)	(n = 854)	
Age (years)		63 ± 8	65 ± 7	63 ± 8	61 ± 9	<0.01
Male (n, %)		1880 (73.41)	572 (66.98)	631 (73.97)	677 (79.27)	<0.01
FBG (mmol/L)		6.41 ± 2.31	5.99 ± 1.95	6.32 ± 2.99	6.92 ± 2.55	<0.01
BMI (kg/m^2^)		25.88 ± 3.35	24.83 ± 3.26	26.07 ± 3.14	26.73 ± 3.37	<0.01
HR (beats/min)		77 ± 12	76 ± 12	77 ± 12	78 ± 13	0.81
SBP (mmHg)		134 ± 18	134 ± 17	135 ± 19	134 ± 18	0.75
DBP (mmHg)		78 ± 12	77 ± 11	78 ± 12	78 ± 12	0.02
HDL-C (mg/dL)		1.03 ± 0.25	1.22 ± 0.25	1.00 ± 0.19	0.86 ± 0.16	<0.01
LDL-C (mg/dL)		2.13 ± 0.82	2.00 ± 0.78	2.15 ± 0.79	2.24 ± 0.86	<0.01
TC (mg/dL)		3.73 ± 0.96	3.63 ± 0.88	3.66 ± 0.91	3.89 ± 1.04	<0.01
TG (mg/dL)		1.48 ± 0.94	0.81 ± 0.21	1.27 ± 0.27	2.35 ± 1.13	<0.01
HbA1c (%)		6.65 ± 1.29	6.42 ± 1.18	6.60 ± 1.26	6.91 ± 1.38	<0.01
eGFR (mL/min)		88.60 ± 16.73	89 ± 14.50	88.17 ± 16.57	88.64 ± 18.86	0.58
Tobacco users (n, %)		1313 (51.27)	374 (43.79)	426 (49.94)	513 (60.07)	<0.01
Alcohol drinkers (n, %)		1692 (66.07)	540 (63.23)	552 (64.71)	600 (70.26)	0.01
Hypertension (n, %)		1752 (68.41)	544 (63.70)	585 (68.58)	623 (72.95)	<0.01
Glucose metabolism state						<0.01
	Non-DM (n, %)	1388 (54.20)	521 (61.01)	476 (55.80)	391 (45.78)	
	DM (n, %)	1173 (45.80)	333 (38.99)	377 (44.20)	463 (54.22)	
Medications						
	Antiplatelets (n, %)	2386 (93.17)	798 (93.44)	795 (93.20)	793 (92.86)	0.89
	Antihypertensive drugs (n, %)	2085 (81.41)	662 (77.52)	700 (82.06)	723 (84.66)	<0.01
	Antilipidemic drugs (n, %)	2388 (93.24)	797 (93.33)	799 (93.67)	792 (92.74)	0.74

FBG, fasting blood glucose; BMI, body mass index; HR, heart rate; SBP, systolic blood pressure; DBP, diastolic blood pressure; LDL-C, low-density lipoprotein cholesterol; TC, total cholesterol; HbA1c, glycated hemoglobin A1c; eGFR, estimated glomerular filtration rate; DM, diabetes mellitus.

Table 2 presents the clinical and demographic characteristics of patients categorized by single-vessel or multivessel CAD. A total of 1990 patients had multivessel CAD. Patients with multivessel CAD were older and predominantly male compared to those with single-vessel disease (all *p* < 0.05). Additionally, the values of AIP, FBG, HDL-C, TC, TG, and HbA1c were higher in patients with multivessel CAD (all *p* < 0.05). They also reported higher proportions of antiplatelet and antihypertensive drug use as well as tobacco use and hypertension (all *p* < 0.05).

**Table 2.** **Baseline clinical and demographic characteristics stratified by CAD severity**.

Variables		Total	Single-vessel CAD	Multivessel CAD	*p* value
		(n = 2561)	(n = 571)	(n = 1990)	
Age (years)		63 ± 8	62 ± 8	63 ± 8	0.05
Male (n, %)		1880 (73.41)	350 (61.30)	1530 (76.88)	<0.01
AIP		0.11 ± 0.27	0.08 ± 0.28	0.12 ± 0.27	0.08
FBG (mmol/L)		6.41 ± 2.31	5.91 ± 1.67	6.55 ± 2.44	<0.01
BMI (kg/m^2^)		25.88 ± 3.35	26.06 ± 3.42	25.83 ± 3.33	0.15
HR (beats/min)		77 ± 12	77 ± 12	77 ± 12	0.40
SBP (mmHg)		134 ± 18	134 ± 18	134 ± 19	0.77
DBP (mmHg)		78 ± 12	78 ± 11	78 ± 12	0.97
HDL-C (mg/dL)		1.03 ± 0.25	1.08 ± 0.26	1.01 ± 0.24	<0.01
LDL-C (mg/dL)		2.13 ± 0.82	2.15 ± 0.78	2.13 ± 0.83	0.47
TC (mg/dL)		3.73 ± 0.96	3.79 ± 0.90	3.71 ± 0.97	0.06
TG (mg/dL)		1.48 ± 0.94	1.47 ± 0.88	1.48 ± 0.96	0.77
HbA1c (%)		6.65 ± 1.29	6.26 ± 1.04	6.76 ± 1.33	<0.01
eGFR		88.60 ± 16.73	91.25 ± 12.95	87.84 ± 17.60	<0.01
Tobacco users (n, %)		1313 (51.27)	244 (42.73)	1069 (53.72)	<0.01
Alcohol drinkers (n, %)		1692 (66.07)	374 (65.50)	1318 (66.23)	0.74
Hypertension (n, %)		1752 (68.41)	361 (63.22)	1391 (69.90)	<0.01
Glucose metabolism state					<0.01
	Non-DM (n, %)	1388 (54.20)	377 (66.02)	1011 (50.80)	
	DM (n, %)	1173 (45.80)	194 (33.98)	979 (49.20)	
Medications					
	Antiplatelets (n, %)	2386 (93.17)	523 (91.59)	1863 (93.62)	0.08
	Antihypertensive drugs (n, %)	2085 (81.41)	437 (76.53)	1648 (82.81)	<0.01
	Antilipidemic drugs (n, %)	2388 (93.24)	529 (92.64)	1859 (93.42)	0.52

### 3.2 Relationship Between AIP and CAD Severity

Higher AIP levels were consistently associated with greater CAD severity across unadjusted and fully adjusted models. Logistic regression outcomes are detailed in Table 3. In Model 1, univariate analysis indicated that the AIP was significantly correlated with CAD severity (OR, 1.667; 95% CI: 1.179–2.356; *p* = 0.004). Compared with the AIP1 group, AIP2 and AIP3 were associated with higher odds (AIP2: OR 1.384, 95% CI: 1.105–1.734; *p* = 0.005; AIP3: OR 1.459, 95% CI: 1.163–1.830; *p* = 0.001). In Model 2, after adjusting for age and sex, AIP, as a continuous variable, was significantly linked to multivessel CAD (OR, 1.770; 95% CI: 1.234–2.540; *p* = 0.002). Subsequent modifications revealed elevated risks of 1.339-fold for the AIP2 group (OR, 1.339; 95% CI: 1.057–1.698; *p* = 0.016) and 1.441-fold for the AIP3 group (OR, 1.441; 95% CI: 1.124–1.848; *p* = 0.004). After adjusting for age, sex, tobacco and alcohol use, SBP, DBP, eGFR, BMI, and the use of antiplatelet, antihypertensive, and antilipidemic drugs, multivariate analysis identified AIP as a continuous variable that remains an independent risk factor for CAD severity (OR, 1.700; 95% CI: 1.160–2.491; *p* = 0.007). Using the AIP1 group as a reference, the AIP2 group exhibited a 1.339-fold increased risk of multi-vessel CAD (OR, 1.339; 95% CI: 1.057–1.698; *p* = 0.016), while the AIP3 group showed a 1.441-fold increase (OR, 1.441; 95% CI: 1.124–1.848; *p* = 0.004).

**Table 3.** **Associations between the AIP and CAD severity**.

Groups	Model 1			Model 2			Model 3		
	OR	95% CI	*p* value	OR	95% CI	*p* value	OR	95% CI	*p* value
AIP	1.667	1.179–2.356	0.004	1.77	1.234–2.540	0.002	1.700	1.160–2.491	0.007
AIP1	Ref.	-	-	Ref.	-	-	Ref.	-	-
AIP2	1.384	1.105–1.734	0.005	1.38	1.091–1.737	0.006	1.339	1.057–1.698	0.016
AIP3	1.459	1.163–1.830	0.001	1.481	1.169–1.876	0.001	1.441	1.124–1.848	0.004

Note: Model 1: Original model. Model 2 is adjusted for both age and sex. Model 3 was adjusted for variables including age, sex, tobacco and alcohol use, systolic and diastolic blood pressure, estimated glomerular filtration rate, body mass index, and the use of antiplatelet, antihypertensive, and antilipidemic medications. OR, odds ratio; CI, confidence interval.

Table 4 presents the relationship between AIP and CAD severity, categorized by diabetes status. In Model 3, with adjustment for age, sex, tobacco and alcohol use, SBP, DBP, eGFR, BMI, and the use of antiplatelet drugs, antihypertensive drugs, and antilipidemic drugs, the AIP2 (OR, 1.415; 95% CI: 1.051–1.904; *p* = 0.022) and AIP3 (OR, 1.421; 95% CI: 1.030–1.962; *p* = 0.033) groups were significantly linked to CAD severity in the non-DM subgroup.

**Table 4.** **Associations between the AIP and CAD severity stratified by diabetes status**.

Diabetes status		Model 1			Model 2			Model 3		
		OR	95% CI	*p* value	OR	95% CI	*p* value	OR	95% CI	*p* value
Non-DM										
	AIP	1.452	0.932–2.263	0.099	1.536	0.964–2.447	0.071	1.521	0.929–2.488	0.095
	AIP1	Ref.	-	-	Ref.	-	-	Ref.	-	-
	AIP2	1.489	1.125–1.971	0.005	1.483	1.114–1.974	0.007	1.415	1.051–1.904	0.022
	AIP3	1.367	1.020–1.833	0.037	1.391	1.025–1.889	0.034	1.421	1.030–1.962	0.033
DM										
	AIP	1.403	0.791–2.490	0.247	1.436	0.793–2.598	0.232	1.464	0.778–2.756	0.237
	AIP1	Ref.	-	-	Ref.	-	-	Ref.	-	-
	AIP2	1.118	0.759–1.647	0.574	1.099	0.740–1.633	0.639	1.114	0.739–1.679	0.607
	AIP3	1.259	0.864–1.835	0.23	1.240	0.837–1.837	0.282	1.228	0.813–1.856	0.328

Note: Model 1: Original model. Model 2 is adjusted for both age and sex. Model 3 was adjusted for variables including age, sex, tobacco and alcohol use, systolic and diastolic blood pressure, estimated glomerular filtration rate, body mass index, and the use of antiplatelet, antihypertensive, and antilipidemic medications.

## 4. Discussion

The study identified a significant link between the AIP and multivessel CAD, suggesting that AIP could be a valuable indicator of CAD severity, even after accounting for clinical risk factors. Notably, this association was stronger in individuals without diabetes.

Recent studies indicate that composite lipid parameters, like the triglyceride–glucose (TyG) index and the AIP, are more effective than individual lipid components in predicting cardiovascular disease risk and mortality [24, 25]. sdLDL is highly susceptible to oxidation, forming oxidized low-density lipoprotein that triggers inflammatory responses in the subendothelial layer of blood vessels, leading to foam cell generation and atherosclerosis [26]. The AIP indicates the balance between triglycerides and HDL-C and is strongly correlated with sdLDL, supporting its predictive capability. Consequently, the AIP can be utilized as an alternative indicator for evaluating sdLDL levels [19, 20]. HDL-C, another characteristic of AIP, is antiatherogenic [27]. Since the AIP can reflect the esterification rate of HDL particles, it offers a more precise assessment of cardiovascular risk than measuring HDL-C alone does [7].

Wu *et al*. [28] employed a case–control design to investigate the subject, identified the AIP as an independent cardiovascular risk factor, consistent with previous studies. A prospective 7.8-year follow-up study involving 52,676 Turkish adults demonstrated that the AIP is a reliable predictor of CAD events and cardiovascular disease (CVD) in both genders. A meta-analysis revealed that elevated AIP values independently correlate with an increased risk of CAD [10]. The underlying mechanism may be related to the consistency between the AIP and plasma atherogenicity. The AIP was linked to atherogenic factors such as obesity, diabetes mellitus, serum uric acid, C-reactive protein, and oxidative stress [11, 14, 29, 30]. These risk factors contribute to CAD development and may explain the high predictive value of the AIP. This study reaffirms the AIP’s efficacy as a biomarker for severe CAD by further investigating its association with CAD severity.

Multivessel CAD is associated with a greater risk of adverse clinical outcomes compared to single-vessel CAD, even following percutaneous coronary intervention (PCI) therapy [31]. Multivessel CAD complicates PCI and indicates the severity of the condition. In this study, higher AIP levels were linked to an elevated risk of multivessel CAD, with AIP identified as an independent predictor of CAD severity. In an observational study that included 1478 young adults with acute coronary syndrome, the AIP was correlated with the number of diseased vessels [32]. Consistent with prior studies, our analysis revealed a strong link between increased AIP and the severity of CAD in a substantial patient cohort. Moreover, on the basis of our observations, the impact of the AIP on multivessel CAD was more pronounced in those without DM. The convergence of findings underscores the AIP’s potential as a biomarker for predicting CVD and suggests that, without diabetes-related metabolic disturbances, the AIP may have a clearer and more precise association with coronary artery lesion development. This may be because, in patients without DM, the absence of DM-related confounding factors on lipid metabolism and vascular function allows the AIP to more accurately represent the mechanisms of atherosclerotic progression. In clinical practice, the AIP may assist in identifying patients at higher risk of extensive coronary disease, particularly among those without diabetes. Future research could focus on exploring the specific pathophysiological pathways through which the AIP plays a predictive role in this particular patient group. Additionally, prospective studies are still necessary to further confirm the clinical utility of the AIP in predicting multivessel coronary artery lesions in patients without DM.

## 5. Limitations

It is important to acknowledge certain limitations of this study. This single-center retrospective study may be subject to selection bias due to the exclusion of numerous patients lacking coronary angiography data, those over 75 years of age, or those meeting other exclusion criteria. Therefore, caution is advised when generalizing these findings to broader populations. Second, the presence of unknown confounding factors, such as variations in education, employment status, and lifestyle habits, which were not captured or analyzed in this study, could influence the observed associations. Future research should focus on overcoming these limitations by conducting larger, diverse prospective studies that thoroughly evaluate a broader spectrum of potential confounders. A limitation of this study is the exclusion of SYNTAX scores, preventing the analysis of their correlation with AIP scores. Future studies are required to clarify the relationship.

## 6. Conclusions

An in-depth analysis demonstrated a significant association between the AIP and the risk of multivessel CAD. This association underscores the potential of the AIP as a valuable marker for predicting disease severity and stratifying risk in patients with CAD.

## Data Availability

The data underlying this study are not publicly available as they include patient-level data, but are available from the corresponding author on reasonable request.

## Abbreviations

AIP, atherogenic index of plasma; CAD, coronary artery disease; BMI, body mass index; eGFR, estimated glomerular filtration rate; HDL-C, high-density lipoprotein cholesterol; TC, total cholesterol; LDL-C, low-density lipoprotein cholesterol; TG, triglyceride; CVD, cardiovascular disease; sdLDL, small dense LDL; DM, diabetes mellitus; CAG, coronary angiography; SBP, systolic blood pressure; DBP, diastolic blood pressure; FBG, fasting blood glucose; HbA1c, glycated hemoglobin A1c; CI, confidence interval; OR, odds ratio; HR, heart rate.

## Supplementary Material

Supplementary material associated with this article can be found, in the online version, at https://doi.org/10.31083/RCM45510.
