# Supplementary material for: Influence of Plasma Atherogenic Index on Coronary Artery Disease Severity: Insights From a Large-Scale Cohort Study in China
Source: Rev Cardiovasc Med. 2026 Apr 14;27(4):45510. doi: 10.31083/RCM45510 (PMC13155972; doi:10.31083/RCM45510)
Supplement: Supplementary file 1 [file 2153-8174-27-4-45510-s1.zip › Supplementary Material.docx]

**Supplementary material**

**Supplementary Methods**

**Measurements of laboratory parameters**

Laboratory parameters, including fasting blood glucose, creatinine, TC, HDL-C, and LDL-C, were measured using the LABOSPECT 008 system (Hitachi, Tokyo, Japan), and glycated hemoglobin A1c (HbA1c) levels were measured using high-performance liquid chromatography (G8, TOSOH, Tokyo, Japan) at the Beijing Hospital laboratory.
